# Functional diversity of Brazilian bees: revealing the unique patterns of the Neotropics

**DOI:** 10.1007/s00442-025-05828-8

**Published:** 2025-12-02

**Authors:** Guaraci D. Cordeiro, Tereza C. Giannini, Patrick M. Consorte, Ana C. J. Costa, Waira S. Machida, Bruno F. Marques, Nicholas D. Mazzei, Poliana P. Menezes, Ludmila S. Resende, Juliana A. Shimoda, Renata S. Souza, André L. Acosta, Antonio J. C. Aguiar, Eduardo A. B. Almeida, Denise A. Alves, Isabel Alves-dos-Santos, Tamires O. Andrade, Evandson J. Anjos-Silva, Alexandre S. Barbosa, Eduardo R. M. Barbosa, Leilane A. Bezerra, Rafael C. Borges, Thaline F. Brito, Gabriela P. Camacho, Alistair J. Campbell, Marina S. Castro, Beatriz W. T. Coelho, Rafael R. Ferrari, Carlos A. Garófalo, Adrian D. González-Chaves, Gabriel O. Keller, Elinor M. Lichtenberg, Leon Marshall, Carlos A. Martínez-Martínez, Marlúcia B. Martins, Aline C. Martins, Márcia M. Maués, Henrique P. Moleiro, Denise M. D. S. Mouga, Favízia F. de Oliveira, Kelli S. Ramos, Ramon L. Ramos, Léo C. Rocha-Filho, Ian P. V. Santos, Samara Santos, José E. Santos Júnior, Akira Shibata, Daniel P. Silva, Fernanda G. Sousa, César M. N. Teixeira, Allison L. Tietz, Matheus E. Trindade-Santos, Patrícia S. Vilhena, Felipe Vivallo, Luísa G. Carvalheiro

**Affiliations:** 1 Universidade Federal de Goiás, Goiânia, 74690-900 Brazil; 2 Instituto Tecnológico Vale, Belém, 66055-090 Brazil; 3 Universidade Federal Rural do Rio de Janeiro, Seropédica, 23890-000 Brazil; 4 University of Oxford, Oxford, OX1 2JD UK; 5 Universidade de Brasília, Brasília, 70910-900 Brazil; 6 Universidade de São Paulo, Ribeirão Preto, 14040-901 Brazil; 7 Universidade de São Paulo, Piracicaba, 13418-900 Brazil; 8 Universidade de São Paulo, São Paulo, 05422-970 Brazil; 9 Universidade Federal do Pará, Belém, 66075-110 Brazil; 10 Universidade do Estado de Mato Grosso, Cáceres, 78216-060 Brazil; 11 Fundação Osvaldo Cruz, Brasília, 70904-130 Brazil; 12 Secretaria de Estado Saúde Pública do Distrito Federal, Brasília, 70719-040 Brazil; 13 Museu Paraense Emílio Goeldi, Belém, 66077-830 Brazil; 14 Museu de Zoologia da Universidade de São Paulo, São Paulo, 04263-000 Brazil; 15 Natural England, Kendal, LA9 7RL UK; 16 Universidade Estadual de Feira de Santana, Feira de Santana, 44230-000 Brazil; 17 University of North Texas, Denton, 76203 USA; 18 Naturalis Biodiversity Center, Leiden, Netherlands; 19 Embrapa Amazônia Oriental, Belém, 66095-903 Brazil; 20 Universidade da Região de Joinville, Joinville, 89219-710 Brazil; 21 Universidade Federal da Bahia, Salvador, 40170-115 Brazil; 22 Universidade Federal de Uberlândia, Uberlândia, 38405-317 Brazil; 23 Instituto Federal de Goiás, Anápolis, 75131-457 Brazil; 24 Universidade Federal do Paraná, Curitiba, 80060-000 Brazil; 25 Universidade Federal do Rio de Janeiro, Rio de Janeiro, 20940-040 Brazil; 26 Instituto de Pesquisa Ambiental da Amazônia, Brasília, 70863-520 Brazil; 27 Amplo Engenharia e Gestão de Projetos, Belo Horizonte, 30140-080 Brazil; 28 Instituto Nacional de Pesquisas da Amazônia, Manaus, 69067-375 Brazil; 29 Jardim Botânico do Rio de Janeiro, Rio de Janeiro, 22460-030 Brazil; 30 Universidade Federal de Minas Gerais, Belo Horizonte, 31270-901 Brazil

**Keywords:** Body size, Buzz, Functional traits, Nesting, Social behavior

## Abstract

Bees are essential for ecosystem functioning, pollinating many wild and crop plant species. Predicting which species are most vulnerable to global changes, and how their loss may impact ecosystems and human well-being, is critical. Comprehensive information on bee response and effect traits is fundamental to these assessments. However, the Raunkiærian shortfall—insufficient trait data—remains significant for bees, particularly in the Neotropics. Moreover, it remains uncertain whether conservation strategies based on functional diversity from temperate regions can be generalized to the Neotropics. To address this gap, we compiled a comprehensive and validated dataset on Brazilian bee traits, covering 24 traits related to sociality (100% of the species with some information), nesting (88%), body size (71%), and buzzing capacity (42%) on 2,066 Brazilian bee species. The trait data here presented is a crucial resource for evaluating bee species' pollination effectiveness and susceptibility to global change. Comparative analysis with regions with ample trait data—USA, Europe, and China—revealed notable differences. Brazilian bees exhibited a higher prevalence of aboveground nesting species, especially compared to Europe. The proportion of eusocial species was also greater than in Europe and the United States, and more similar to China. Differently from other regions, Brazilian eusocial bees were significantly smaller than their non-eusocial counterparts. These cross-regional comparisons highlight the importance of geographically tailored conservation strategies and underscore the need for extensive trait data to accurately predict regional vulnerabilities and ecological impacts in a rapidly changing world.

**Supplementary Information:**

The online version contains supplementary material available at 10.1007/s00442-025-05828-8.

## Introduction

Species traits influence how organisms interact with their environment and with other species (Hooper et al. 2005; Violle et al. 2007; Schleuning et al. 2015), thus affecting their adaptation, survival, and fitness (response traits), as well as their contribution to ecosystem functioning (effect traits) (Williams et al. 2010). Indeed, knowledge of species traits is essential for defining ecological niches (McGill et al. 2006), explaining geographic ranges (Aguirre-Gutiérrez et al. 2016), evaluating the impacts of ongoing global changes (Henn et al. 2018; Carvalheiro et al. 2020; Giannini et al. 2020), and improving assessments of ecosystem service provision (Costanza et al. 1997; McGill et al. 2006; Elizalde et al. 2020). However, compiling and standardizing species trait data is often challenging and time-consuming, requiring a careful search through various sources, including published papers, postgraduate theses and dissertations, open-access databases, museum specimens, and even fieldwork notes. Such scarcity of information about species traits and their ecological functions, i.e., the Raunkiæran shortfall (Hortal et al. 2015), is more accentuated and challenging to address in tropical regions due to their mega-diversity (Jablonski et al. 2006; Mittelbach et al. 2007; Brown 2014). As a result, it is unclear what patterns are found in these regions and how functionally similar species assemblages are from other regions of the world.

Bees are crucial organisms for the provision of ecosystem functions by pollinating most flowering plants (Potts et al. 2016; Ollerton 2017). They also play an important role in agricultural production and the global economy (Giannini et al. 2015; Oliveira et al. 2024). Characterizing the functional diversity of this group in a given region can help inform strategies to conserve wild populations under anthropogenic pressures, enhance the prediction of pollination services, and support the development of more sustainable agricultural practices. However, studies on bee traits and their functions are still limited (Martins et al. 2015; Campbell et al. 2022; Chase et al. 2023), resulting in a lack of general predictions from trait-based approaches (but see Bartomeus et al. 2018; Coutinho et al. 2018). In certain regions with a long-standing tradition of bee research, such as Europe (Leclercq et al. 2023) and the USA, information on traits is accumulating (Harmon-Threatt 2020; Orr et al. 2022; Marshall et al. 2024), enabling a deeper understanding of anthropic impacts on bee diversity and consequently, ensuring more informed conservation and management strategies. For example, studies in European cities have shown that bees benefit from high proportions of impervious surfaces at large scales, regardless of whether bees are below or aboveground nesters (Weber et al. 2023). However, a study conducted in Brazil found no relationship between the percentage of impervious surfaces and nesting stratum, but reported that generalist bees, particularly highly eusocial species, benefit from higher proportions of impervious surfaces, whereas specialist bees do not (Gomes et al. 2025). These contrasting results suggest that the functional composition of the bee fauna often differs between temperate and tropical regions, limiting the potential for generalizations regarding conservation strategies. Therefore, there is an urgent need to obtain global standardized and referenced information on bee traits, with a greater focus on understudied regions, such as the Neotropics (Ostwald et al. 2024).

Here, we focused on Brazilian bees, which represent a large proportion of the known Neotropical fauna (Moure et al. 2023), as a proxy for functionally characterizing Neotropical bees. Brazil occupies over 40% of the Neotropical region (Morrone et al. 2022) and is home to approximately 10% of all described bee species globally (Ascher and Pickering 2024), a high percentage that probably reflects the continental dimension of the country, its vast latitudinal range, and the diversity of landscapes (from lowlands to mountain ridges, and savannas to rainforests) encompassed by several biodiversity hotspots (e.g., *Cerrado*, Atlantic and Amazon forests) (Myers et al. 2000). We focused on four groups of traits widely recognized as ecologically relevant, since they influence how bees respond to environmental changes (response traits) and affect ecosystem functioning (effect traits): sociality, nesting, body size, and buzzing capacity. The degree of sociality in bees is associated with their foraging range, communication strategies, and cooperation among colony members (Nieh 2004; Michener 2007; Leonhardt 2017; Elizalde et al. 2020; Grüter and Hayes 2022; Kendall et al. 2022; Alves et al. 2023). Body size is a significant predictor of foraging range, food requirements, temperature tolerance, and pollination efficiency (Araújo et al. 2004; Greenleaf et al. 2007; Stang et al. 2009; Pinto et al. 2015; McCabe et al. 2019; Raiol et al. 2021; Kendall et al. 2022; Castillo et al. 2023). Nesting traits encompass suitable sites, substrates, and specific behaviors related to nest construction and protection, as well as strategies to deter attacks by natural enemies, and influence population-level sex ratios (Moure-Oliveira et al. 2017; Harmon-Threatt 2020). Finally, buzzing capacity is associated with the use of power thoracic muscle vibration to remove pollen grains from poricidal anthers, influencing both foraging behavior and pollination efficiency in certain plant families, such as the economically important Solanaceae (Buchmann 1983; Nunes-Silva et al. 2013; Rosi-Denadai et al. 2020; Vallejo-Marín 2022).

The availability of information on these traits is, therefore, instrumental to facilitating bee research. For instance, bee trait data has contributed to our understanding of how bees respond to land-use conversion and intensification (De Palma et al. 2015; Coutinho et al. 2018; Buchholz and Egerer 2020), provided insights into predictions regarding climate change (Giannini et al. 2020; Maebe et al. 2021), and the crucial role that functional traits diversification plays in pollination services (Campbell et al. 2022; Chase et al. 2023). As tropical bees have a longer period of activity than their temperate counterparts, it is likely that strategies requiring high resource investments, such as advanced levels of sociality, are more common in tropical regions than in temperate ones. Indeed, the evolution and distribution of eusociality among bees is likely associated with warmer temperatures (Brady et al. 2006). Moreover, as larger body sizes help bees survive better in colder regions (Maebe et al. 2021), bees tend to be smaller in tropical than in temperate areas. Sociality level can also influence thermoregulatory tasks of bees, such as fanning and regulating brood temperature (Jones et al. 2004; Weidenmüller 2004). Therefore, climate-driven evolution pressures may have led to distinct proportions of specific traits in different bee communities. To test these expectations, we compared the functional composition of Brazilian bees with that of bees from other parts of the world. As a result of this work, we herein launch the Brazilian Bee Trait Database (henceforth, BBTD), a collaborative initiative by multiple Brazilian research groups aimed at gathering and curating a comprehensive trait-based dataset for Brazilian bee species to address the Raunkaerian shortfall in Neotropical bees.

## Material and methods

The BBTD was built based on data from 2066 bee species native to Brazil (Supplementary Table S1). Of these, 1965 were included in the current version of Moure’s Catalogue of Bees (Hymenoptera, Apoidea) in the Neotropical region (Moure et al. 2023), and 101 species were included in our database after a taxonomic literature review of the recent works. From the total number of native species of Brazil, 1114 (54.1%) belonged to Apidae, 350 (16.9%) to Megachilidae, 347 (16.8%) to Halictidae, 133 (6.4%) to Colletidae, and 122 (5.9%) to Andrenidae. Two introduced bee species in Brazil, *Anthidium manicatum* (Linnaeus, 1758) and *Lithurgus huberi* Ducke, 1907 (Strange et al. 2011; Silva et al. 2014; Graf et al. 2020), were also included in our species list.

To provide a more comprehensive description of species traits, a consensus-based approach was applied to major categories of sociality (eusocial *vs.* non-eusocial), nesting (above *vs.* below ground), and buzzing ability (yes *vs*. no). Whenever all species with available information within a given subgenus, genus, tribe, or subfamily exhibited the same trait, suggesting trait conservatism at the taxonomic level, we assigned that same trait to all its constituent species with missing information. For nesting traits this approach was applied for ‘Nesting broad classification’ (above *vs.* below ground), ‘Nesting Method’ and ‘Nesting Substrate’. We also recorded the supraspecific taxonomic levels at which each inference was made to reflect the associated level of accuracy. This procedure has been considered a robust approach because all bee taxa listed herein comprise monophyletic groups according to the available phylogenomic evidence (Almeida et al. 2023). Furthermore, all classifications were carefully revised by bee experts specialized in each group, all of whom are authors of this work. Both our genus- and species-level classifications followed Moure et al. (2023); however, we used the family-level classification of Michener (2007), including modifications proposed in recent years (summarized in Almeida et al. 2025).

### Focal traits

The current version of BBTD comprises four key groups of bee traits: sociality, nesting, body size, and buzzing capacity. Each group of traits has one or more categories with subcategories (Supplementary Table S2).

The broadest class of sociality considered here divides bee species into two main categories, eusocial and non-eusocial, each with its subcategories, defined as in Michener (2007). Eusocial species exhibit caste differentiation, reproductive division of labor, age polyethism, and generational overlap. This category includes the three subcategories: highly eusocial bees (e.g., *Trigona spinipes* (Fabricius, 1793)), primitively eusocial bees (e.g., *Euglossa townsendi* Cockerell, 1904), and cleptobiotic social species that invade nests of other bees to steal food (e.g., *Lestrimelitta limao* (Smith, 1863)). Non-eusocial species are divided into four subcategories: they either nest alone (solitary, e.g., *Tetrapedia diversipes* Klug, 1810), share nests without cooperation (communal, e.g., *Cephalurgus anomalus* Moure and Lucas de Oliveira, 1962), form short-lived colonies with reproductive division of labor (semisocial, e.g., *Xylocopa frontalis* (Olivier, 1789)), or lay eggs in brood cells of other species (cleptoparasitic, e.g., *Coelioxys simillima* Smith, 1854). For the two main categories (eusocial *vs*. non-eusocial), the consensus approach was used across all families, except for two genera, *Augochloropsis* and *Euglossa*, where there is frequent occurrence of mixed social behaviors within these groups.

The broadest class of nesting divides bees into species that nest either “above ground”, “below ground”, or both below and above ground. Aboveground species were then classified into four subcategories depending on whether they use pre-existing cavities (e.g., within trunks), build exposed nests (surfaces of tree trunks or walls), excavate their own nest (e.g., in termite mounds), or use a diverse set of these strategies. They were also classified based on whether they used urban structures or not. Belowground species establish nests below ground level (e.g., *Geotrigona mombuca* (Smith, 1863)). Those nesting below ground were divided into only two subcategories: excavate or use pre-existing cavities.

Body size is measured as the intertegular distance (ITD), a widely used proxy for body size (Cane 1987). Albeit continuous, ITD was divided into three categories as proposed by Borges et al. (2020) for standardization purposes: small (≤ 2.2 mm, e.g., *Tetragonisca angustula* (Latreille, 1811)), median (2.21–3.9 mm, e.g., *Megachile susurrans* Haliday, 1836), and large (≥ 3.91 mm, e.g., *Bombus brasiliensis* Lepeletier, 1836). Information was divided between males and females (also considering differences between workers and queens in social bees), for which mean values obtained for the species are presented. In addition, we generated a metric with a general indicator of the mean value per species. For most species, such a metric was the mean value of the females. Yet, for some Euglossini species for which we only had information on males, we used the value of males as an indicator of a comparative size metric. For some species in this tribe (e.g. *Exaerete* and *Aglae*), females tend to be slightly larger than males, so this approach might underestimate size (Roubik 2019). However, for other species within the group, there are no significant differences in size between males and females (Peruquetti 2003; Medina et al. 2016).

Buzzing capacity refers to the ability of bees to vibrate their thoracic muscles to remove and collect pollen and maximize its efficiency of foraging on distinct flower morphologies (e.g., *Oxaea flavescens* Klug, 1807) (De Luca and Vallejo-Marin 2013; Russel et al. 2024) and is categorized as a binary trait (yes *vs.* no).

These categories were derived from previous studies, which in turn proposed them according to their standard protocols or methodologies. The authors’ expertise also guided the decisions on category definitions and the interpretation of available data. Several other important traits, such as dietary specialization and pilosity pattern, were left out of the scope of this work, but they remain a priority for inclusion in future updates of the BBDT.

### Literature search

Trait information was obtained through a systematic literature search that included articles related to Brazilian bees and their functional traits using the Web of Science (Clarivate Analytics) and Google Scholar. The search involved combining species names, commonly used synonyms, and functional trait terms in both English and Portuguese. For example, to obtain data on body size, the following query was used: (*Trigona spinipes*) AND (body size OR body length OR intertegular distance OR ‘*tamanho do corpo’* OR ‘*distância intertegular’*) AND (Brazil OR *Brasil*). This process was repeated for each of the 2066 bee species native to Brazil. When a relevant paper was identified, its cited references were also examined for additional sources.

The search yielded 529 studies containing trait information. Among these, 89% were peer-reviewed research papers, 6% were Master’s or Doctoral theses/dissertations, 4% were books or book chapters, and 1% were conference papers.

For each trait added to the database, we included information about its source and the corresponding level of taxonomic resolution (e.g., tribe, genus, species). Trait entries at the species level were only assigned when published studies provided specific information for the corresponding species. For broader taxonomic levels (e.g., genus or tribe), a consensus-based approach was applied: a trait was attributed to an entire group only if the majority of species within that group exhibited it, based on available data. This process was further reviewed and validated by specialists with expertise in the respective bee group. The consensus-based approach was only applied to traits that reflected broad descriptions, such as nesting behavior (above *vs.* below ground), sociality (eusocial *vs.* non-eusocial), and buzzing capacity (yes *vs.* no).

### Body size measurements

We complemented the bee body size information extracted from the literature with ITD measurements. We focused on species for which no records were extracted from the literature and focused on female individuals (workers in the case of social bees), trying to reach a minimum of five individuals per species. Yet, for Euglossini (Apidae: Apinae), we gathered information on males for several species because they are better represented in museum collections.

### Comparison of functional trait distribution across regions

Even though this work is focused on Brazilian bees, we also selected bee trait databases from other regions of the world for a comparative discussion of trait distribution (Supplementary Table S3). More specifically, we searched for databases covering large spatial regions, such as entire (sub)continents or major parts of them, as Brazil (8,510,000 km^2^) does for South America, which, when combined, led to a list of at least 1000 species per region. We identified three regions that matched our selection criteria: Europe (covered area ca. 7,150,000 km^2^), USA (9,867,000 km^2^), and China (9,597,000 km^2^). The European database, as referenced in Marshall et al. (2024) (derived from the ‘European Bee Traits Database’, originally created by ALARM, www.alarm-project.ufz.de; further developed by STEP, www.STEP-project.net; and currently curated and updated by S.P.M. Roberts), comprises information on sociality, nesting, and ITD across 2,137 species. For the USA (3,333 species) and China (1,174 species), we extracted trait information from several publications that compiled data from either the target region or for bees worldwide (for a list of references, see Supplementary Table S3). To provide more comprehensive information on the major description of sociality (eusocial *vs*. non-eusocial), the consensus-based approach (described above) was applied to the bee data from all regions (see details in Supplementary Table S3). We used the set of species for which there was available information and then estimated the proportion of species that are classified as eusocial *vs*. non-eusocial and as nesting below *vs*. above ground. For comparisons of ITD between social classes, for each region, we used a generalized linear model assuming a Gamma distribution (log link function) with sociality class as the explanatory variable. As a sensitivity test, we reran analyses using only species for which we had species-level accuracy on trait information. All analyses were done with software R (R Core Team 2024).

## Results

We obtained data on at least one trait for all 2,066 bee species listed for Brazil, totaling 13,746 trait entries. Information on sociality was available for 2,066 species (100%), nesting biology for 1,824 (88%), buzzing capacity for 1,455 (71%), and body size for 857 (42%) species.

**Fig. 1:**
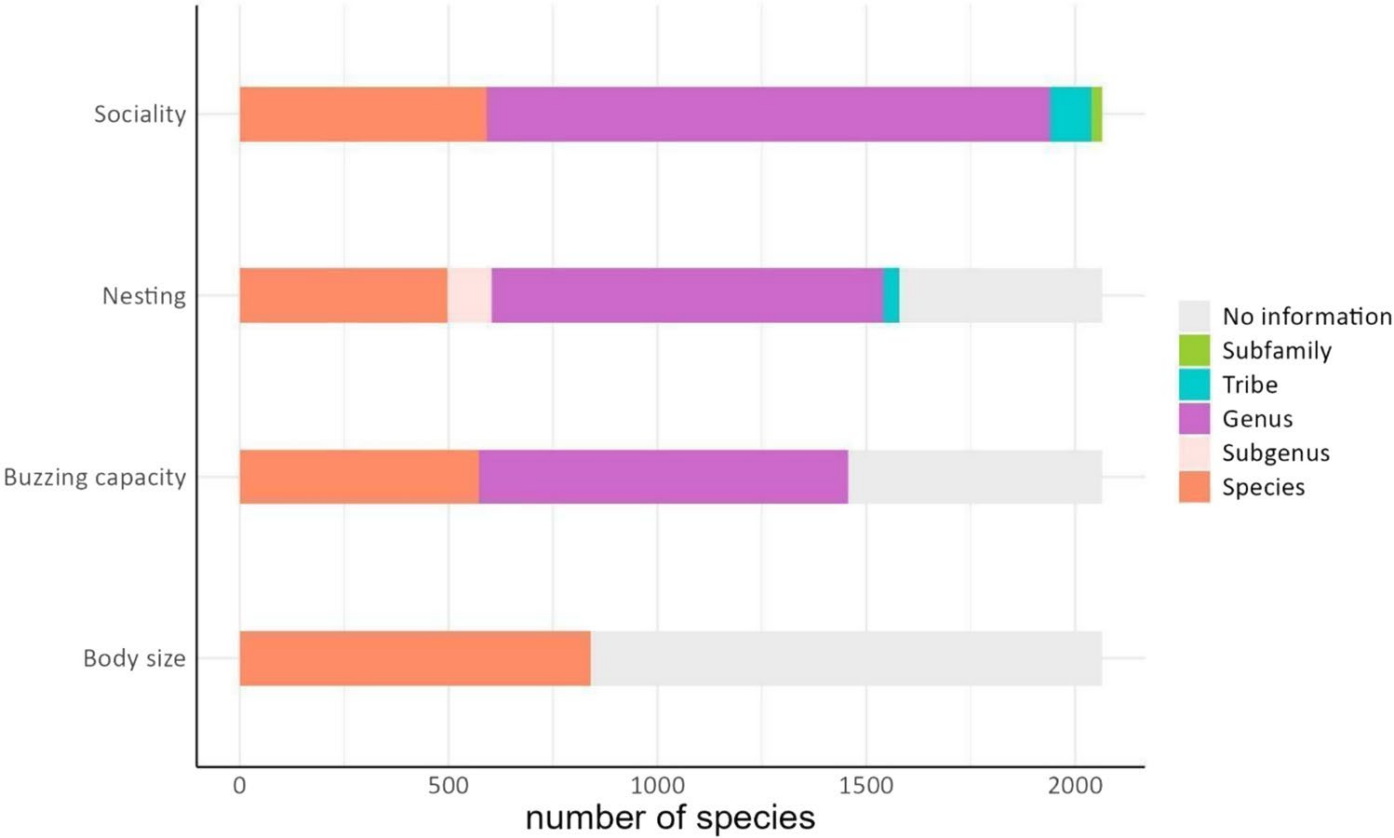
Number of species with information at different levels of accuracy for each broad class within the four bee trait groups. For sociality, this refers specifically to eusocial *vs.* non-eusocial; for nesting, it refers to aboveground *vs*. belowground nesting

The accuracy of the recorded information varied among the traits. For sociality, the accuracy of the data was 28% of the trait entries were based on information at the species level, 66% at the genus level, 4% at the tribe level, and 1% at the subfamily level (Neopasiphaeinae, Colletidae). It is important to highlight that for the sociality broad class descriptions (eusocial vs. non-eusocial) consistency typically occurs within tribe or even family (1665 species). Only for the tribes Augochlorini and Euglossini is there variability within tribe, and consensus needs to be achieved within genus based on species with well-described sociality patterns. Only for two specific genera (*Augochloropsis*, *Euglossa*) was there variability within genus. While these two genera are well known for being composed of non-eusocial species, there are exceptional and well-described cases of eusociality. For these two genera, all species that had no information on sociality, were assumed to be non-social. For nesting, the accuracy of the data was: species—28%, genus—60%, subgenus—5%, tribe—2%. For buzzing capacity, we obtained information for 804 species (39%), and for the remaining 1,257 species (61%), entries were inferred based on data from congeneric ones. Body size measurements were exclusively obtained at the species level (Fig. 1).

### Sociality

**Fig. 2:**
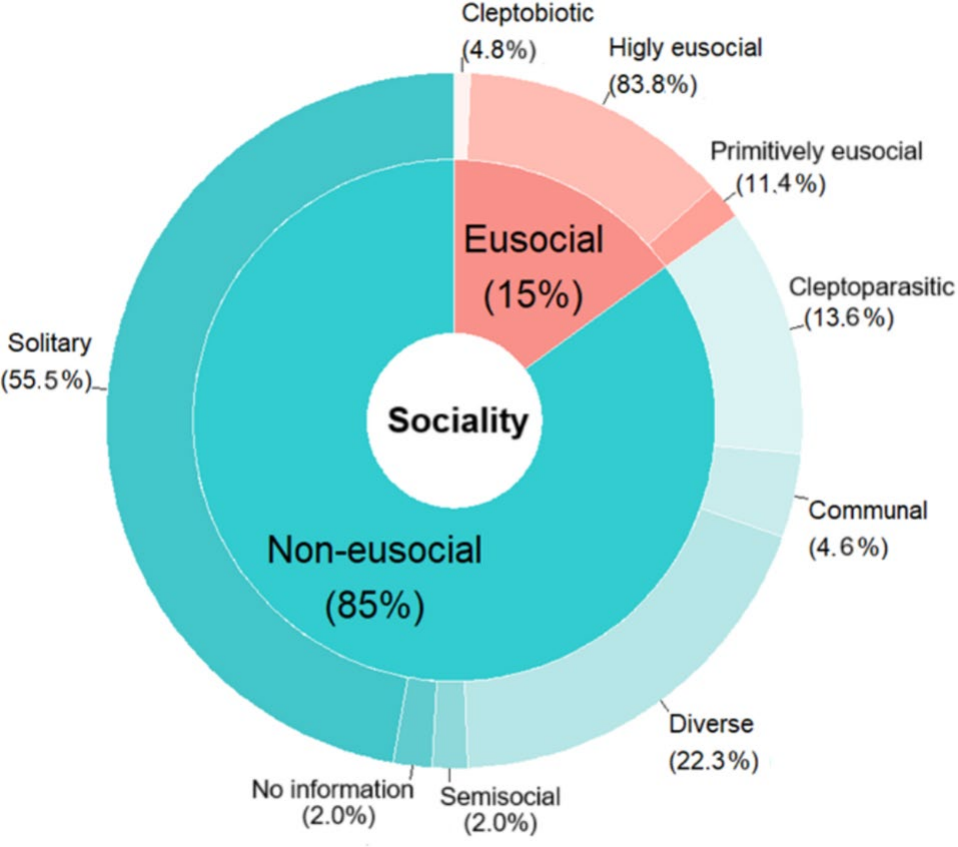
Frequency of each sociality level and sublevels among 2,066 Brazilian bee species with information available. Diverse: non-eusocial species with more than one type of social behavior. Percentage of taxonomic level of trait assignments: Subfamily 1.2%; tribe 4.8%; genus 65.3,%; species 28.7%

Most bee species in Brazil (1,752 species, 85%) are non-eusocial, while eusocial ones represent 15% (309 species) of the total. Within the non-eusocial category, most species are solitary (55.5%) or exhibit some level of sociality (communal or semisocial, 30.9%), while 13.6% are cleptoparasitic; for the latter, we obtained data on host associations for only 48 species (20%). Among eusocial species, only 4.8% exhibit a cleptobiotic behavior (Fig. 2).

### Nesting

**Fig. 3:**
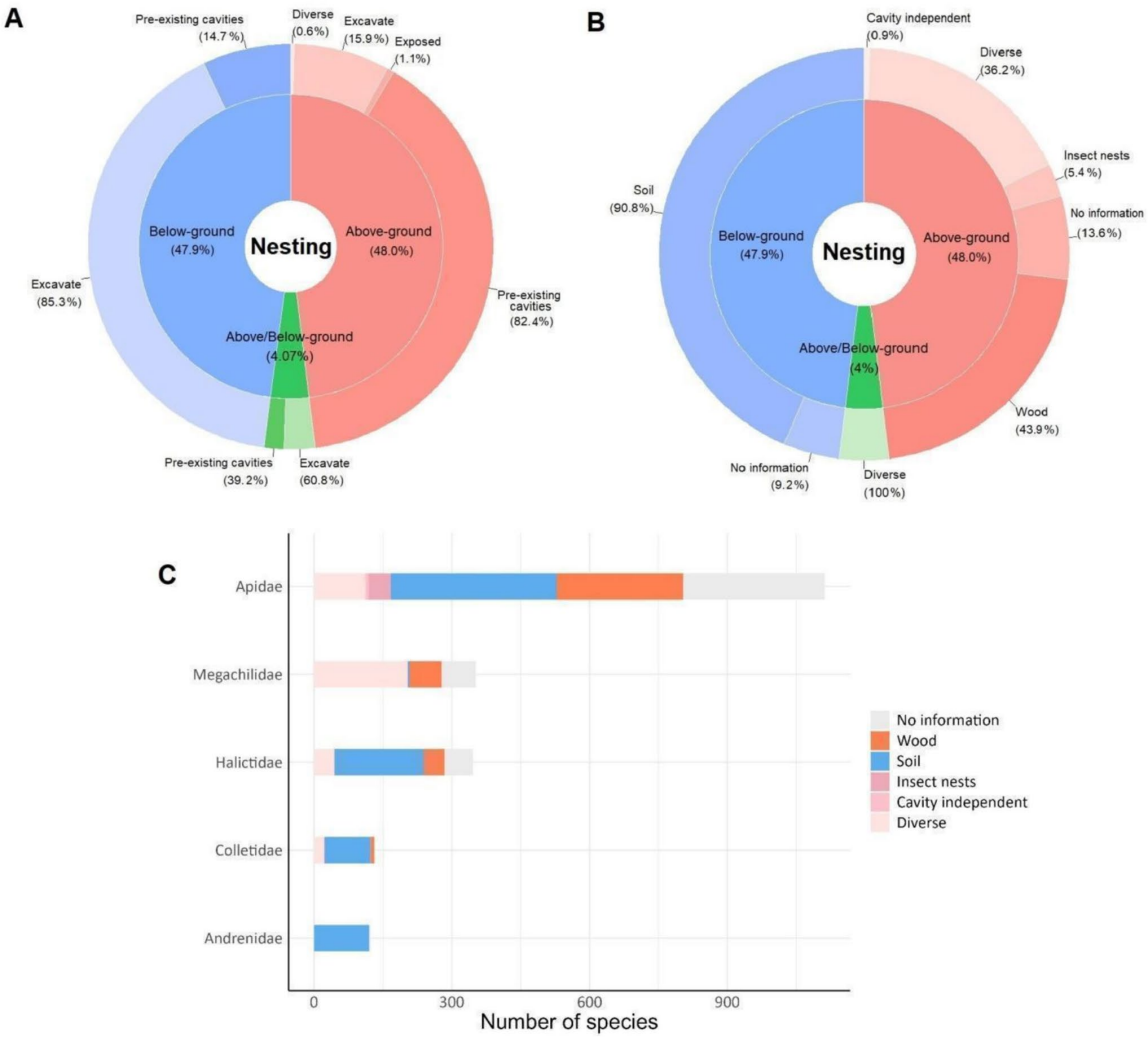
Overview of the available bee nesting traits data. **A** Percentage of above- and belowground nesting bees distributed by nesting method; diverse: species able to both excavate and use pre-existing cavities. **B** Percentage of above- and belowground nesting bees distributed by specific substrate; diverse: species that can use more than one type of nesting substrate. **C** The number of species with different substrate preferences within each bee family. Percentage of taxonomic level of trait assignments: tribe 2.3%; genus 59.5%; subgenus 6.7%; species 31.5%

We obtained information from 1,824 species (88%), of which 876 (48%) nest above ground, 874 species (47.9%) nest below ground, and 74 species (4.1%) nest both above and below ground. Among the belowground nesters, most (93.9%) establish their nests through excavation, while most (86.9%) aboveground nesters use pre-existing cavities (Fig. 3A). Furthermore, aboveground nesters primarily utilize wood (43.9%) or use more than one type of substrate (36.2%) to build a nest (Fig. 3B). Most species nest in the soil regardless of the family they belong to, except Megachilidae species, which use mostly wood and human structures to establish their nests (Fig. 3C).

Among the species that nest above ground, 312 (15%) also build nests in human-made structures, such as fence posts, roofs, and walls. Most of these species (95%) built nests in pre-existing wooden cavities. We also found that at least 363 bee species in Brazil (16%) use artificial nests, such as artificial hives and trap nests. These species belong to Megachilidae (189 species, 52%), Apidae (169 species, 47%), and Colletidae (5 species, 1%). Of these, 279 species (77%) also use human-made structures; 271 species of them were found nesting in trap nests, and 150 belong to the genus *Megachile*. Of the species kept in artificial hives (92 species) most belong to the genus *Melipona* (35 species).

**Fig. 4:**
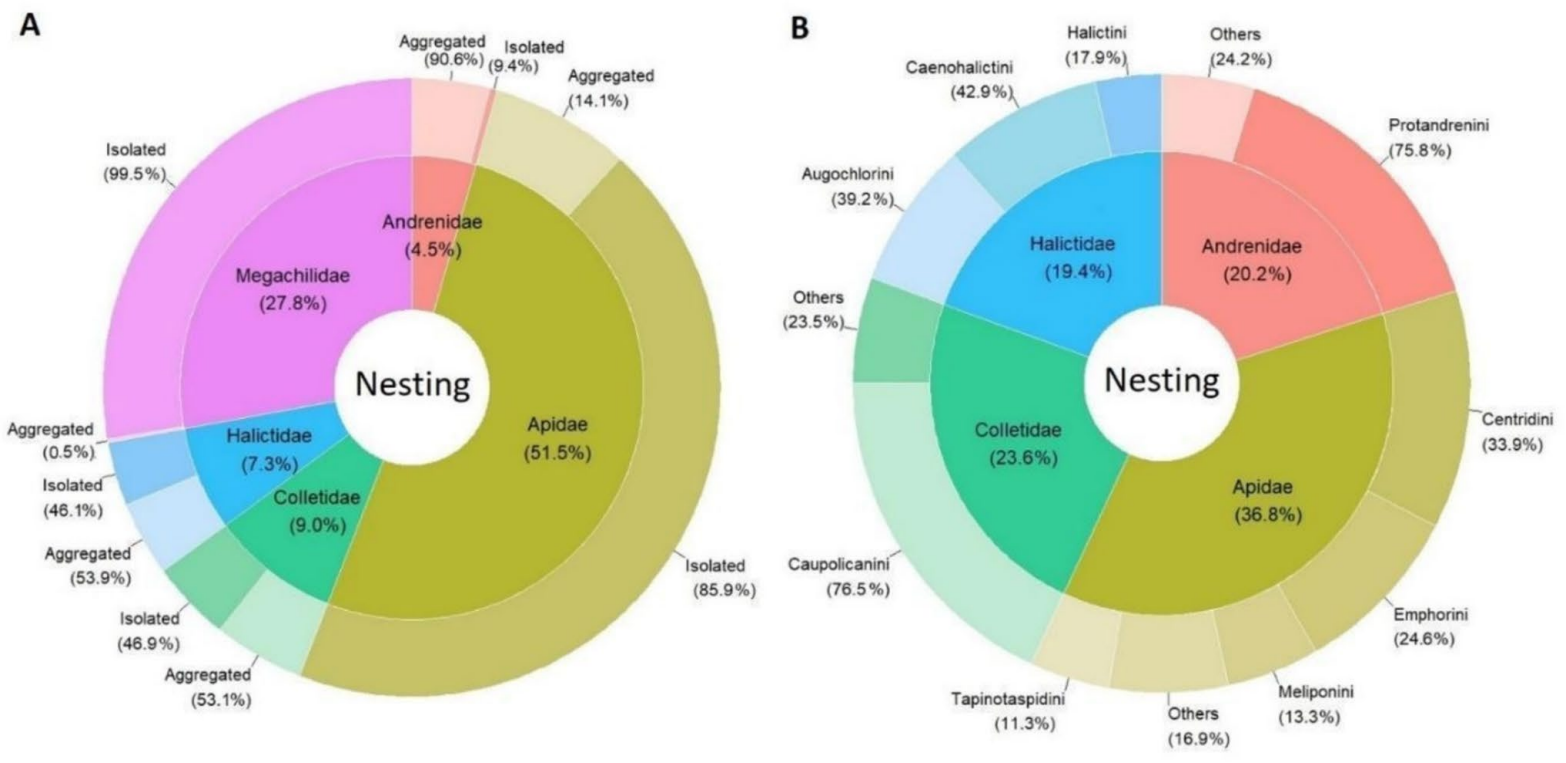
Overview of bee nest-building behavior. **A** Percentage of species with different behaviors per family. **B** Percentage of species with aggregation behavior within families and tribes. Percentage of taxonomic level of trait assignments: tribe 0.4%; genus 41%; subgenus 2.8%; species 55.8%

We found information about species nesting in aggregations (or not) for 753 species, representing 37% of the total. This included cleptoparasitic species, for which we used information on their known hosts. Females of 586 species (78%) did not exhibit aggregation behavior and were found to build only isolated nests, while 167 species (22%) built aggregated nests. Additionally, 22 cleptoparasitic species parasitize these aggregating hosts. Apidae and Megachilidae included the largest proportion of species that built isolated nests (85.9% and 99.5%, respectively), while most species of the other families showed aggregated behaviour (Fig. 4A). Among the species with aggregated nests, Apidae has the highest percentage (36.8%, mostly Centridini), followed by Colletidae (23.6%, mostly Caupolicanini), Andrenidae (20.2%, mostly Protandrenini), and Halictidae (19.4%, mostly Caenohalictini) (Fig. 4B); no megachilid species were found to exhibit such behavior.

We compiled data on colony size (i.e., number of workers) and nest architecture (i.e., brood cell arrangement) for 79 stingless bee species (Meliponini) from 23 genera. The genera with the highest number of individuals per nest were *Trigona* and *Ptilotrigona* (Supplementary Figure S1). Brood cell arrangements varied among genera: multilayered horizontal combs in *Cephalotrigona*, *Geotrigona*, *Lestrimelitta*, *Melipona*, *Paratrigona*, *Partamona*, *Ptilotrigona*, *Scaptotrigona*, and *Tetragonisca*; semi-combs in *Duckeola* and *Friesella*; and clusters in *Celetrigona*, *Frieseomelitta*, *Leurotrigona*, and *Trichotrigona*. *Nannotrigona*, *Oxytrigona*, *Schwarziana*, and *Tetragona* exhibit combs or spiral combs, while *Plebeia* and *Scaura* may construct horizontal or spiral combs, or even cluster arrangements. *Trigona* builds horizontal, semi, and spiral combs. The comb of *Scaura longula* (Lepeletier, 1836) has a vertical orientation, being the only stingless bee species known to possess this arrangement.

### Body size

**Fig. 5:**
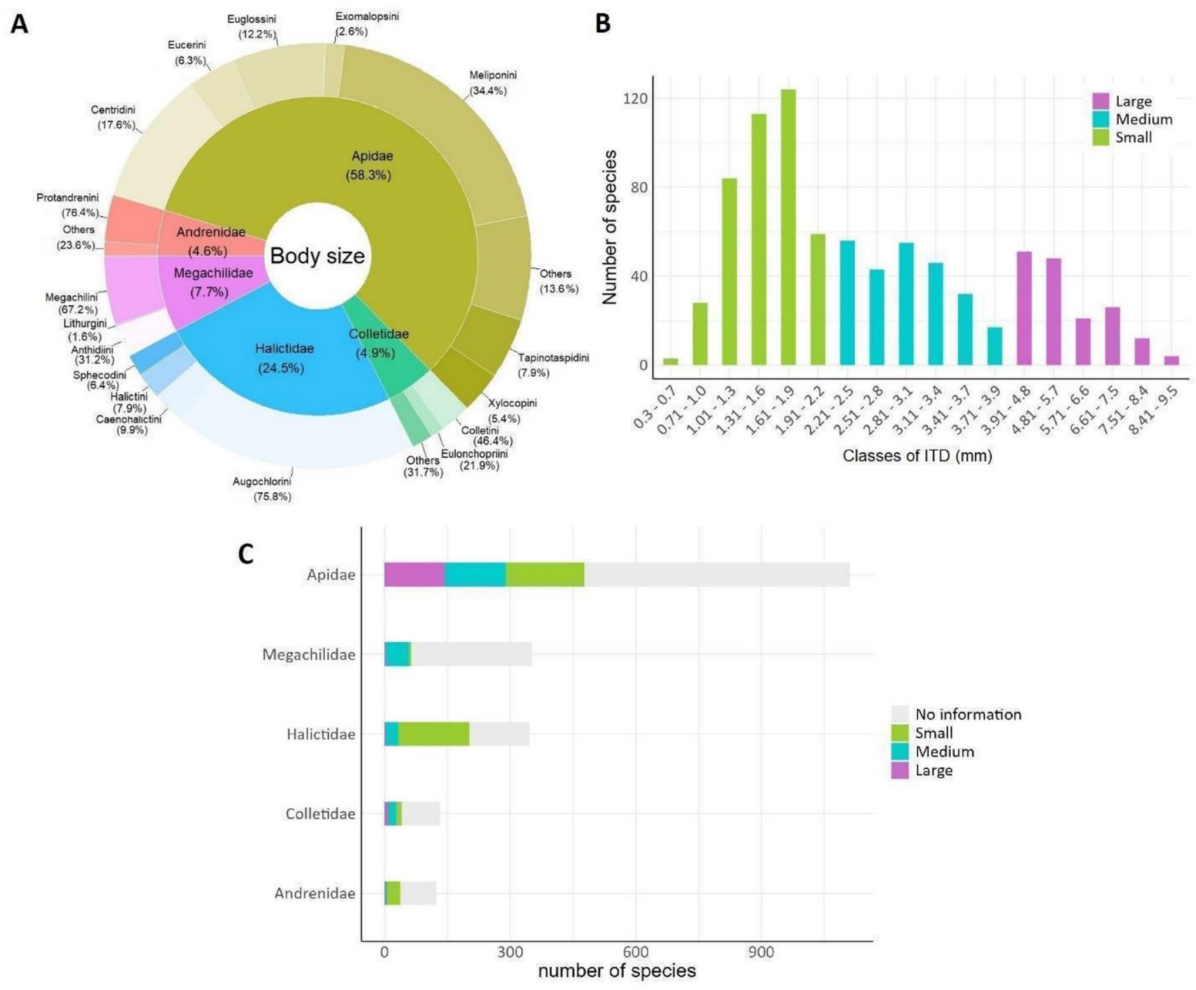
Overview of bee body size data based only on females (workers for social bees). **A** Percentage of species with available data within bee families and tribes, **B** number of species within body size intervals and classes of intertegular distance (ITD): small (≤ 2.20 mm), medium (2.21–3.90 mm), and large (≥ 3.91 mm), **C** distribution of body size classes within bee families

We obtained information on intertegular distances (ITD) for 857 Brazilian bee species, accounting for 42% of the described bee species in Brazil. The data were primarily from species of Apidae (58.3%) and Halictidae (24.5%) (Fig. 5A). Despite comprising 17% of the Brazilian bee fauna, Megachilidae corresponded to only 7.7% of the species for which ITD data were available. Meliponini (34.4%) and Centridini (17.6%) accounted for most of the available ITD data within Apidae. Augochlorini (75.8%) contributed the most to the data available for Halictidae.

Of the 857 bee species for which we obtained information on body size, the average ITD was 2.77 ± 1.71 mm. Overall, eusocial species showed a significantly smaller ITD (1.77 ± 0.84 mm, *n* = 173) compared to non-eusocial ones (3.02 ± 1.78 mm, *n* = 684) (Chi = 109.04; *p* < 0.001). Most of the bee species (51%) found in Brazil were small (≤ 2.2 mm) (Fig. 5B). This same pattern was observed for Apidae, Halictidae, and Andrenidae. However, Megachilidae and Colletidae included mostly medium-sized species (2.21–3.9 mm) (Fig. 5C).

### Buzzing capacity

**Fig. 6:**
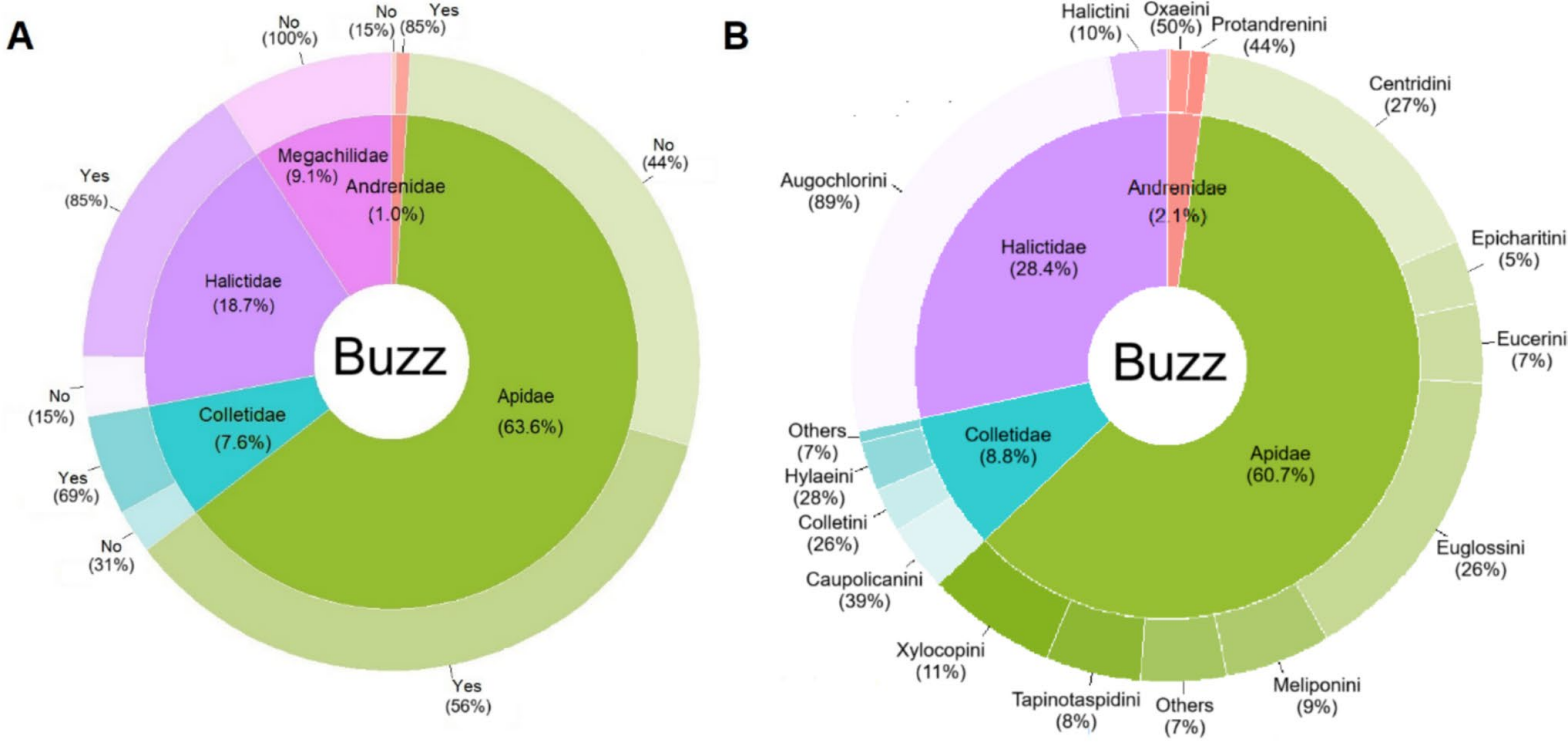
Overview of data concerning the buzzing behavior of Brazilian bee species across bee families (**A**) and their tribes (**B**). Graphs were built based only on species for which we had information available. Percentage of taxonomic level of trait assignments: genus 60.6%; species 39.4%

We obtained information on buzzing capacity for 1,455 species, accounting for 71% of the bee species described in Brazil. Among these, 843 species (58%) were capable of buzzing. The highest percentages of buzzing behavior were observed in Halictidae (85%), Andrenidae (85%), and Apidae (56%), while no instances were recorded for Megachilidae (Fig. 6A). Most buzzing species in Halictidae, Andrenidae, and Apidae belong to Augochlorini (89%), Oxaeini (50%), and Centridini (27%), respectively (Fig. 6B).

### Comparison of functional trait distribution across regions

**Fig. 7:**
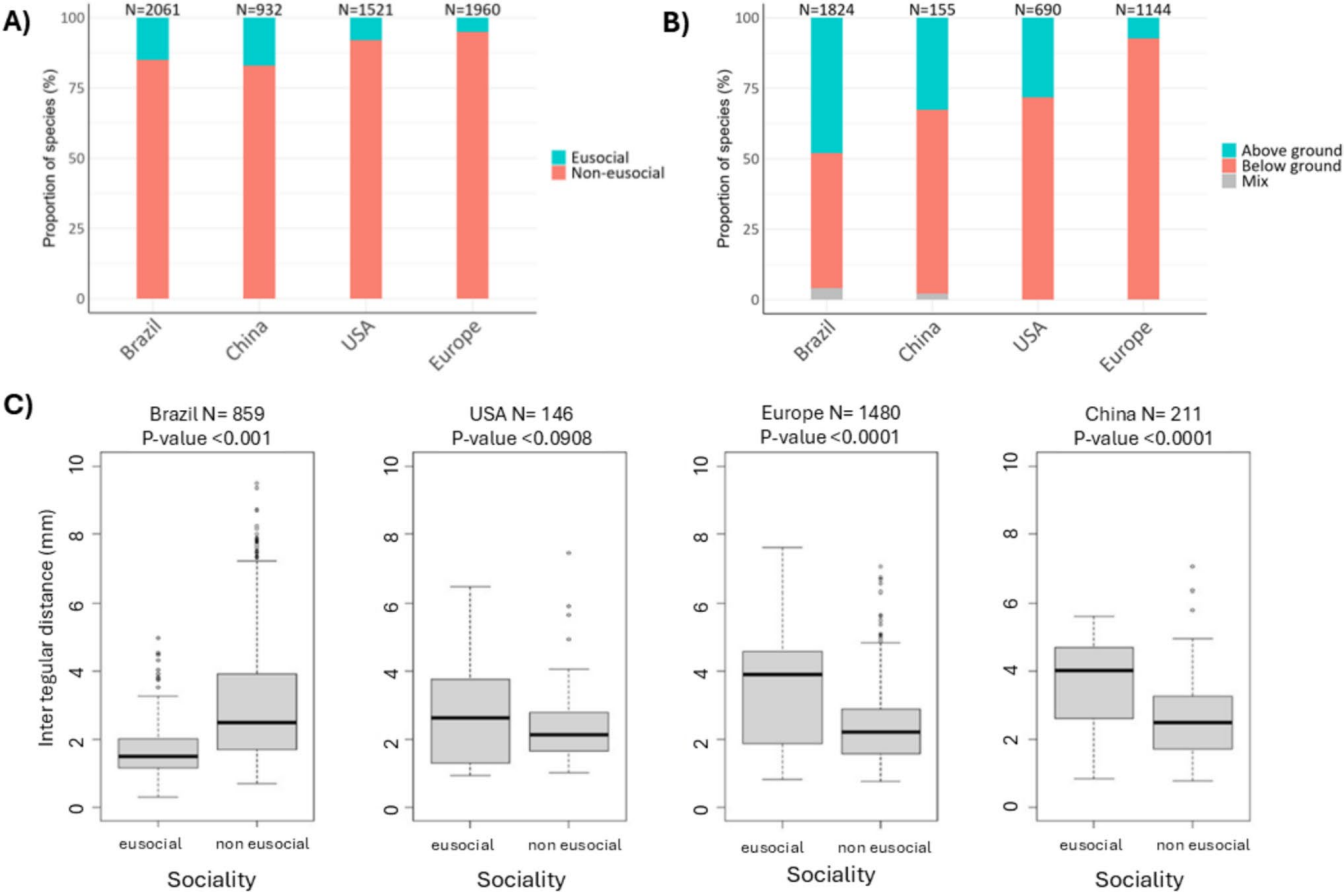
Comparison of bee trait distribution across regions. For sociality (**A**) and nesting (**B**), we only used the broadest categorization (eusocial *vs.* non-eusocial; above *vs*. below ground). Comparisons of ITD are provided in **C** for each sociality class. *P*-values presented were obtained with a log-likelihood ratio test

Information on sociality was available for 77% of Chinese bees, 45% of the bees from the USA, and 91% of the European bee species. Nesting information was available for 13% of the Chinese, 21% of the American, and 54% of the European species. Body size information was obtained for 19%, 4%, and 74% of the species from these regions, respectively. Comparative analyses revealed that the proportion of eusocial species in Brazil (15%) is similar to that found in China (17%), but much higher than that found in Europe (5%) and the USA (4%) (Fig. 7A). The proportion of eusocial bees in Brazil became even more distinct from other regions in the sensitivity analyses done with only species that had species-level information on traits (Supplementary Table S4). More strikingly, the Brazilian fauna showed a higher prevalence of aboveground nesters (48% of the 1824 species with nesting information) compared to China (29%, *n* = 155), the USA (28%, *n* = 690) and Europe (7%, *n* = 1144) (Fig. 7B), and this is even more evident at the species level (Supplementary Table S5).

The average ITD of Brazilian bees was not significantly different from that of Chinese bees, but it was significantly higher than that of both European and American species (Supplementary Table S6). Moreover, significant differences were detected regarding specific functional groups (Fig. 7C) even when analyzed only with species at the species level trait information (Figure S2). In both Europe and China, eusocial species were significantly larger than non-eusocial ones, a trend also detected in the USA, where the number of species with available information was very low. In contrast to other regions, Brazilian eusocial species showed a significantly smaller body size than their non-eusocial counterparts.

## Discussion

Trait-based approaches are necessary to address the urgent need to understand how environmental changes affect biodiversity and ecosystem functioning (e.g., Rumeu et al. 2018; Campbell et al. 2022; Xie et al. 2023). The demand for trait-based information to support the development of sustainable management strategies for bee-mediated crop pollination has never been so high (Martins et al. 2015; Woodcock et al. 2019; Laha et al. 2020). Our work represents a significant effort to collate trait information on Brazilian bees (over 13,000 data entries representing 2,066 species), and to reduce the Raunkiærian shortfall, thereby advancing tropical bee research. However, the remaining gap is still substantial, since data are still missing on at least one major trait for 90% of the Brazilian bee species. In contrast, this gap affects “only” 68% of European species (Marshall et al. 2024). Moreover, experts agree that the number of undescribed species is likely higher in the tropics, and the current estimates suggest that less than two-thirds of the actual number of Brazilian bee species are known (Moure et al. 2023).

Although the BBTD will be regularly updated with newly described species and additional trait information, some trends in data completeness (or scarcity) are worth highlighting. For instance, coverage is currently higher for sociality and nesting traits than for body size and buzzing capacity. These two latter traits typically require species-level information; however, they can be easily extracted from detailed morphological characterizations provided in the original species descriptions. We, therefore, urge bee taxonomists to continue providing high-quality, scaled images in future taxonomic publications, and to include average intertegular distance (ITD) values for both males and females, as these data are essential for extracting information on traits highly relevant to predicting bee responses to environmental changes, particularly body size and flight capacity. This will become particularly powerful as computer vision AI models capable of extracting measurements from images advance.

Similarly to the general bee pattern, most Brazilian bee species are non-eusocial. The fact that Brazil has approximately twice as many eusocial species as the USA and Europe can be attributed to the ubiquitous presence of stingless bees (Apidae: Meliponini) in the Neotropics. In tropical environments, higher levels of social organization are often favored due to year-round availability of floral resources, relatively stable temperatures, and a greater diversity of ecological interactions. These factors may have promoted the evolution of perennial colonies and social complexity (Biesmeijer and Slaa 2006; Grüter 2020). In contrast, temperate regions are characterized by more pronounced seasonal fluctuations, which impose constraints that tend to favor solitary or semi-social life strategies (Michener 1974); however, some eusocial taxa can be highly successful in these conditions (e.g., *Bombus*). Resource scarcity during colder months limits the viability of perennial colonies, as continuous foraging becomes unfeasible (Goulson 2010; Cameron and Sadd 2020). As a result, many bee species in these regions exhibit annual life cycles with predominantly solitary or primitively social behaviors. These patterns underscore the critical role of climatic and ecological factors in shaping sociality in bees, highlighting the evolutionary divergence between tropical and temperate lineages and their distinct strategies for colony organization and reproduction. The fact that China, predominantly a temperate country with a broad range of Köppen climate classes, has a proportion of social bees similar to that found in Brazil was unexpected. However, sociality information was available for only 906 Chinese bee species, which represents less than half of the actual bee diversity of the country (Ascher and Pickering 2023). It is thus possible that there are even greater knowledge shortfalls for non-eusocial bees, or that existing data are disproportionately concentrated in warmer regions of China. A more comprehensive assessment of bee sociality in China is needed to confirm the actual distribution of social behavior among bees in this part of the world (Warrit et al. 2023).

Another major difference among Brazilian bees lies in their nesting habits. On a global scale, nearly three-quarters of bee species nest below ground, a behavior considered the ancestral state for bees (Debevec et al. 2012; Sann et al. 2018; Antoine et al. 2021). Unlike the dominance of belowground nesting observed on other continents, Brazilian bee species exhibit an almost equal distribution between belowground (48.0%) and aboveground (47.9%) nesting. In contrast, 93% of European bee species nest below ground (Marshall et al. 2024), as do 72% in North America (Harmon-Threatt 2020; Ferrari and Polidori 2022; Buckner et al. 2024) and 68% in Asia (Xie et al. 2023). This distinct nesting pattern has relevant implications for bee conservation. Bee communities relying more heavily on aboveground nesting are particularly vulnerable to habitat loss, particularly deforestation, which reduces the availability of suitable nesting sites, especially for stingless bees, which rely on hollow trees as protected cavities for nesting (Brown and Oliveira 2014; Grüter 2020; but see Lichtenberg et al. 2017; Campbell et al. 2022). Recent studies have shown that the rate of adaptation of tropical forests to face the climate crisis has been slower than needed, and that large, dense-wooded trees (which typically shelter the most bee nests in Brazil) are the most affected (Aguirre-Gutiérrez et al. 2025a; 2025b; Gonzalez-Chaves et al. 2025). Similarly, belowground nesting bees may also be negatively affected by habitat loss, particularly due to urbanization and intensive agriculture, which reduce the availability of exposed, undisturbed soil required for nesting (Harmon-Threatt 2020; Tschanz et al. 2025). Further studies on the functional diversity of bees in other tropical regions would help clarify the relationship between nesting preferences and climate. Ultimately, the patterns of sociality and nesting distribution among Brazilian bees may reflect their relatively early diversification and ecological adaptations within the Neotropics (Michener 1978; Almeida et al. 2023).

We observed a significant prevalence of larger bees in Brazil and China compared to the USA and Europe. This pattern is unexpected, as Bergmann’s Rule (Bergmann 1847) suggests that larger body sizes are typically advantageous in colder climates rather than in warmer ones. Although studies on insects have yielded conflicting results, some research indicates that Bergmann’s Rule may hold true among closely related species (Osorio-Canadas et al. 2016; Gérard et al. 2018; Suni and Dela-Cruz 2021). In contrast, eusocial species in Brazil, which are predominantly represented by stingless bees, exhibited a significantly smaller body size than their non-eusocial counterparts, a pattern opposite to that observed in other regions (Ferrari and Polidori 2022; Xie et al. 2023; Buckner et al. 2024; Marshall et al. 2024).

The proportion of bee species capable of buzzing in Brazil (58%) is consistent with expectations for tropical bees and is higher than that observed in non-tropical regions (Russell et al. 2024). The greater diversity of buzzing bees in tropical regions most likely reflects the coevolution between these bees and a high variety of plants with poricidal anthers in these environments (Cardinal et al. 2018; Vallejo-Marin and Russell 2024; Russell et al. 2024). These plant-bee interactions are fundamental to maintaining the high productivity of a wide range of agroecosystems across Brazil (Giannini et al. 2015; Cooley and Vallejo-Marín 2021).

By providing an overview of functional traits in Brazilian bees, this study highlights substantial differences in trait patterns across global regions. Therefore, generalizations based on findings from other regions, such as population declines due to deforestation or climate change, should be made with caution (Saunders et al. 2020). The detailed species-level information provided in BBTD can also support future research on the impacts of climate change on plant-pollinator interactions in different regions of Brazil, as well as the development of policies and management actions aimed at protecting this important group of species. Future studies focusing on traits should prioritize bee taxa identified by BBTD as having limited or no available data. For instance, bee families with limited data available at the species level (e.g., Andrenidae) require special attention to fill the knowledge gaps. We also encourage future studies to investigate the role played by other key bee traits, such as tongue length (Miller-Struttmann et al. 2015; Maebe et al. 2021), hairiness (Stavert et al. 2016; Maebe et al. 2021), type of pollen-carrying structures (Weinman et al. 2023), flight phenology (Dorian et al. 2023), and diet breadth (Roulston and Cane 2000; Maebe et al. 2021). BBTD will be a valuable tool for providing information and helping identify research topics where further investigations might be needed. Further steps should also include database expansion to integrate species from other Neotropical countries.

## Supplementary Information

Below is the link to the electronic supplementary material.

Supplementary file1 (DOCX 307 KB)

Supplementary file2 (XLSX 457 KB)

Supplementary file3 (XLSX 611 KB)

## Data Availability

The data will be stored in GitHub (https://github.com/lgcarvalheiro/BBTD) and also in a database accessible via the official website from UFG (https://colecaozoologica.icb.ufg.br/p/brazilianbeetraitdatabase), which will be regularly updated.

## Code availability

Not aplicable.
