# Correction: Functional diversity of Brazilian bees: revealing the unique patterns of the Neotropics

**DOI:** 10.1007/s00442-025-05852-8

**Published:** 2026-01-08

**Authors:** Guaraci D. Cordeiro, Tereza C. Giannini, Patrick M. Consorte, Ana C. J. Costa, Waira S. Machida, Bruno F. Marques, Nicholas D. Mazzei, Poliana P. Menezes, Ludmila S. Resende, Juliana A. Shimoda, Renata S. Souza, André L. Acosta, Antonio J. C. Aguiar, Eduardo A. B. Almeida, Denise A. Alves, Isabel Alves-dos-Santos, Tamires O. Andrade, Evandson J. Anjos-Silva, Alexandre S. Barbosa, Eduardo R. M. Barbosa, Leilane A. Bezerra, Rafael C. Borges, Thaline F. Brito, Gabriela P. Camacho, Alistair J. Campbell, Marina S. Castro, Beatriz W. T. Coelho, Rafael R. Ferrari, Carlos A. Garófalo, Adrian D. González-Chaves, Gabriel O. Keller, Elinor M. Lichtenberg, Leon Marshall, Carlos A. Martínez-Martínez, Marlúcia B. Martins, Aline C. Martins, Márcia M. Maués, Henrique P. Moleiro, Denise M. D. S. Mouga, Favízia F. de Oliveira, Kelli S. Ramos, Ramon L. Ramos, Léo C. Rocha-Filho, Ian P. V. Santos, Samara Santos, José E. Santos Júnior, Akira Shibata, Daniel P. Silva, Fernanda G. Sousa, César M. N. Teixeira, Allison L. Tietz, Matheus E. Trindade-Santos, Patrícia S. Vilhena, Felipe Vivallo, Luísa G. Carvalheiro

**Affiliations:** 1 Universidade Federal de Goiás, Goiânia, 74690-900 Brazil; 2 Instituto Tecnológico Vale, Belém, 66055-090 Brazil; 3 Universidade Federal Rural do Rio de Janeiro, Seropédica, 23890-000 Brazil; 4 University of Oxford, Oxford, OX1 2JD UK; 5 Universidade de Brasília, Brasília, 70910-900 Brazil; 6 Universidade de São Paulo, Ribeirão Preto, 14040-901 Brazil; 7 Universidade de São Paulo, Piracicaba, 13418-900 Brazil; 8 Universidade de São Paulo, São Paulo, 05422-970 Brazil; 9 Universidade Federal do Pará, Belém, 66075-110 Brazil; 10 Universidade do Estado de Mato Grosso, Cáceres, 78216-060 Brazil; 11 Fundação Osvaldo Cruz, Brasília, 70904-130 Brazil; 12 Secretaria de Estado Saúde Pública do Distrito Federal, Brasília, 70719-040 Brazil; 13 Museu Paraense Emílio Goeldi, Belém, 66077-830 Brazil; 14 Museu de Zoologia da Universidade de São Paulo, São Paulo, 04263-000 Brazil; 15 Natural England, Kendal, LA9 7RL UK; 16 Universidade Estadual de Feira de Santana, Feira de Santana, 44230-000 Brazil; 17 University of North Texas, Denton, 76203 USA; 18 Naturalis Biodiversity Center, Leiden, Netherlands; 19 Embrapa Amazônia Oriental, Belém, 66095-903 Brazil; 20 Universidade da Região de Joinville, Joinville, 89219-710 Brazil; 21 Universidade Federal da Bahia, Salvador, 40170-115 Brazil; 22 Universidade Federal de Uberlândia, Uberlândia, 38405-317 Brazil; 23 Instituto Federal de Goiás, Anápolis, 75131-457 Brazil; 24 Universidade Federal do Paraná, Curitiba, 80060-000 Brazil; 25 Universidade Federal do Rio de Janeiro, Rio de Janeiro, 20940-040 Brazil; 26 Instituto de Pesquisa Ambiental da Amazônia, Brasília, 70863-520 Brazil; 27 Amplo Engenharia e Gestão de Projetos, Belo Horizonte, 30140-080 Brazil; 28 Instituto Nacional de Pesquisas da Amazônia, Manaus, 69067-375 Brazil; 29 Jardim Botânico do Rio de Janeiro, Rio de Janeiro, 22460-030 Brazil; 30 Universidade Federal de Minas Gerais, Belo Horizonte, 31270-901 Brazil

**Correction: Oecologia (2025) 208:5** 10.1007/s00442-025-05828-8

In the ‘Material and Methods’ section, the statement which currently read incorrectly as “The European database compiled by Marshall et al. (2024) (derived from the ‘European Bee Traits Database’, originally created by ALARM, www.alarm-project.ufz.de; further developed by STEP, www.STEP-project.net; and currently curated and updated by S.P.M. Roberts), comprises information on sociality, nesting, and ITD across 2,137 species.”

but should have been

“The European database, as referenced in Marshall et al. (2024) (derived from the ‘European Bee Traits Database’, originally created by ALARM, www.alarm-project.ufz.de; further developed by STEP, www.STEP-project.net; and currently curated and updated by S.P.M. Roberts), comprises information on sociality, nesting, and ITD across 2,137 species.”

In the Acknowledgements section, the statement which currently read incorrectly as “The European database, as referenced in Marshall et al. (2024) (derived from the ‘European Bee Traits Database’, originally created by ALARM, www.alarm-project.ufz.de; further developed by STEP, www.STEP-project.net; and currently curated and updated by S.P.M. Roberts), comprises information on sociality, nesting, and ITD across 2,137 species.”

but should have been

“We thank to Stuart Roberts for providing access to the raw bee traits data for Europe.”

The original article has been corrected.

