# Supplementary material for: Functional diversity of Brazilian bees: revealing the unique patterns of the Neotropics
Source: Oecologia. 2025 Dec 2;208(1):5. doi: 10.1007/s00442-025-05828-8 (PMC12672835; doi:10.1007/s00442-025-05828-8)
Supplement: Supplementary file 1 — Supplementary file1 (DOCX 307 KB) [file 442_2025_5828_MOESM1_ESM.docx]

**Supplementary Information**

**Functional diversity of Brazilian bees: revealing the unique patterns of the Neotropics**

**Table S1.** Brazilian Bee Trait Database. Comprehensive dataset of functional traits for Brazilian bee species, including information on sociality, nesting behavior, body size, and buzzing capacity. Metadata describing column content and the references used to extract trait information are provided in associated spreadsheets. (See complementary .xlsx file)

**Table S2.** Trait terminology used in the Brazilian Bee Trait Database and the respective categories in which the data were organized. The levels of sociality were defined as in Michener (2007)^1^. The nesting categories were determined according to the authors' expertise. The classes of arrangements of brood cells followed Grüter (2020)^2^. We use intertegular distance as a proxy of body size following Cane (1987)^3^, and the body size classes are those proposed by Borges et al. (2020)^4^.

| **Sociality^1^** |  |  |
| --- | --- | --- |
| Level of sociality | Eusocial | Long-lived colonies exhibit highly social behavior characterized by morphological differences between castes, reproductive division of labor, age polyethism, and overlap of generations (including truly eusocial, primitively eusocial and cleptobiotic – invade the nests of other species to steal stored food). |
|  | Non-eusocial | One female establishes and maintains her own nest (solitary). Multiple females inhabit the same nest but do not cooperate (communal) or small and short-lived colonies with reproductive division of labor among females (semi-social and primitively eusocial). Females lay their eggs in the brood cells built by females of another bee species (cleptoparasitic). |
| **Nesting** |  |  |
| Position | Above ground | Establish nests above the ground level. |
|  | Below ground | Establish nests below the ground level. |
| Method | Pre-existing cavities | Build nests within pre-existing holes in flat ground, banks, wood, abandoned nests, or human-made structures. |
|  | Excavate | Build nests by excavating burrows in flat ground, banks, or wood. |
|  | Exposed | Build nests in exposed substrates. |
| Substrate | Soil | Establish nests in various types of soil (e.g., earthen, sandy, rocky). |
|  | Wood | Establish nests within tree trunks and branches, plant stems, and twigs. |
|  | Other insect nests | Establish nests within other insect nests (e.g., termites, ants, or wasps). |
|  | Human-made structures | Establish nests in structures built by humans (e.g., fences, roofs, walls). |
|  | Cavity independent | Establish exposed nests, which are made with plant material, mud, or other materials, built on tree branches and human-made structures. |
| Aggregation |  | Establish nests close to each other (yes/no). |
| Colony size |  | Number of adult workers per nest in eusocial species. |
| Brood cell^2^ | Clusters | Brood cells are arranged in clusters held together by thin cerumen connections. |
|  | Combs | Horizontally stacked parallel layers, supported by a central pillar, consisting of brood cells that open upwards. |
|  | Vertical-combs | One layer of brood cells opens upwards. |
|  | Semi-combs | Brood cells are connected in groups but arranged in an irregular pattern. |
|  | Spirals | Brood combs are arranged in a helicoidal pattern without the support of a central pillar. |
| Artificial nests |  | Establish nests in bee hives or trap nests. |
| **Body Size** |  |  |
| Intertegular distance (ITD) ^3,4^ | Small | ≤ 2.2 mm |
|  | Medium | 2.21–3.9 mm |
|  | Large | ≥ 3.91 mm |
| **Buzzing capacity** |  |  |
| Buzz |  | Capacity of body vibration to remove and collect pollen (yes/no) |

**Table S3.** Data used for comparative analyses of sociality (eusocial vs. non-eusocial), nesting (above vs. below ground) and inter-tegular distance between Brazil, USA, Europe and China. For comparison purposes, for broad-scale comparisons of sociality all social parasites were considered eusocial. The consensus approach was applied to complete sociality information (whenever all of the species within a genus that had information had the same trait, that trait was assigned to species with missing information). Metadata describing the content of each column and the references used to extract information on each trait are provided in associated spreadsheets. (See complementary .xlsx file)

**Table S4 -** Proportion of eusocial and non-eusocial species across regions when applied the consensus approach (All), solely with species level information and without data of dubious genus.

|  | **All** | | **Species level** | | **W/o dubious genus** | |
| --- | --- | --- | --- | --- | --- | --- |
|  | **Eusocial** | **Non-eusocial** | **Eusocial** | **Non-eusocial** | **Eusocial** | **Non-eusocial** |
| Brazil | 0.150 | 0.850 | 0.402 | 0.598 | 0.159 | 0.841 |
| China | 0.165 | 0.835 | 0.163 | 0.837 | 0.167 | 0.833 |
| Europe | 0.053 | 0.947 | 0.058 | 0.942 | 0.053 | 0.947 |
| USA | 0.040 | 0.960 | 0.139 | 0.861 | 0.040 | 0.960 |

**Table S5 -** Proportion of nesting broad classification (above vs. below ground) across regions when applied the consensus approach (All) and solely with species level information.

|  | **All** | | | **Species level** | | |
| --- | --- | --- | --- | --- | --- | --- |
|  | **Above** | **Below** | **Above and below** | **Above** | **Below** | **Above and below** |
| Brazil | 0.480 | 0.479 | 0.041 | 0.713 | 0.287 | - |
| China | 0.395 | 0.601 | 0.004 | 0.348 | 0.644 | 0.007 |
| Europe | 0.133 | 0.867 | 0.001 | 0.129 | 0.871 | - |
| USA | 0.199 | 0.801 | - | 0.296 | 0.704 | - |

**Table S6.** Statistical details of the evaluation of differences in body size (intertegular distance) across broad sociality classes and regions. Inter-tegular distance (ITD) data were analyzed with a generalized linear model (Gamma distribution with log link function) with 'region' as an explanatory variable, followed by a Tukey's Honest Significant Difference (HSD) test. For each comparison, the estimate of the difference between the two regions and associated standard error (SE) are provided, as well as the HSD test and P value.

| **Comparison** | **Estimate** | **SE** | **z-value** | **P-value** |
| --- | --- | --- | --- | --- |
| China−Brazil | -0.01123 | 0.03717 | -0.3 | 0.9896 |
| Europe−Brazil | -0.16801 | 0.02108 | -8.0 | < 0.001 |
| USA−Brazil | -0.13557 | 0.04412 | -3.1 | 0.0101 |
| Europe−China | -0.15678 | 0.03536 | -4.4 | < 0.001 |
| USA−China | -0.12434 | 0.05247 | -2.4 | 0.0754 |
| USA−Europe | 0.03244 | 0.04261 | 0.8 | 0.863 |

**
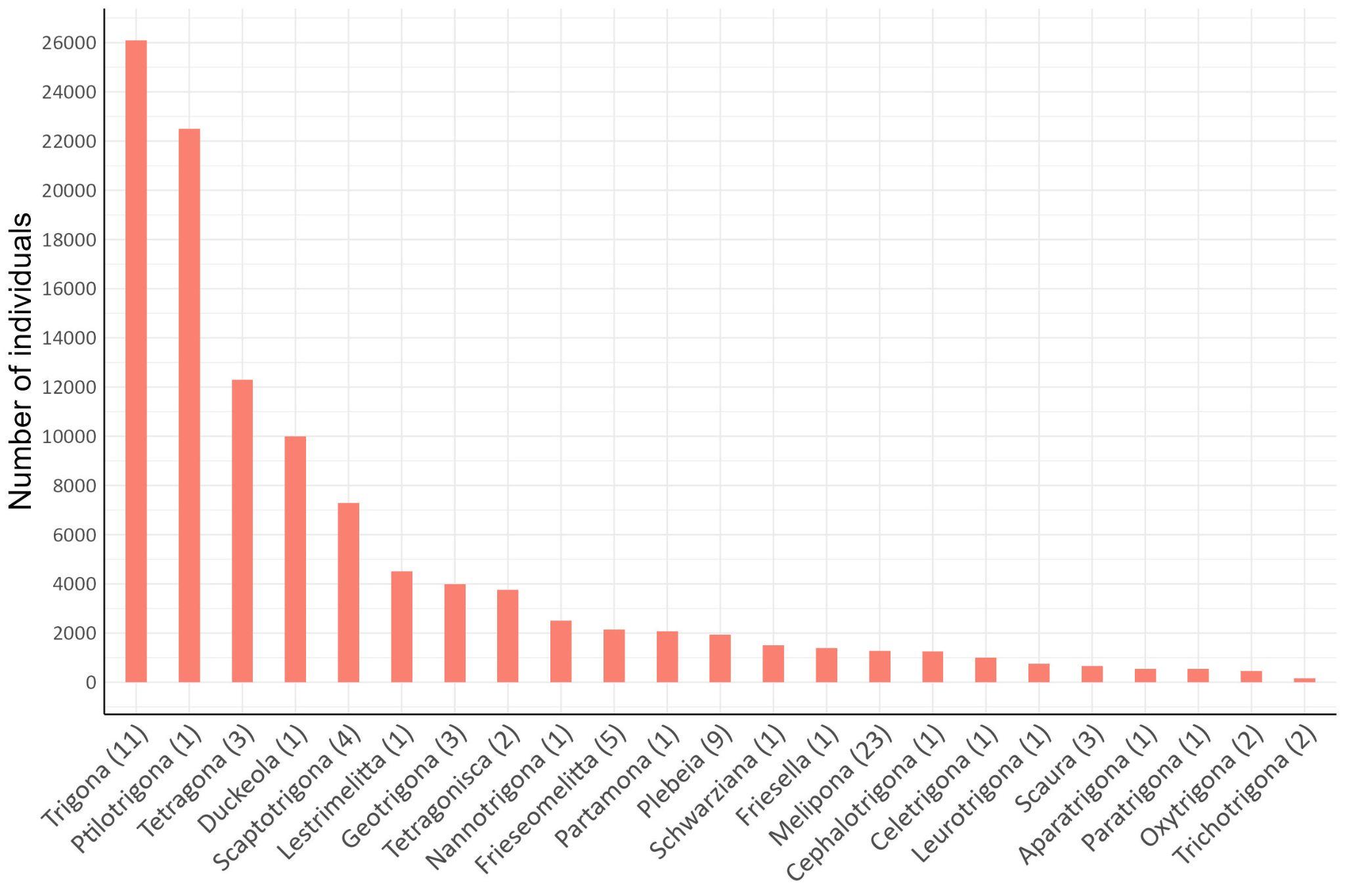
**

**Figure S1.** Overview of colony size (mean number of individuals) within Meliponini. The numbers in parentheses indicate the number of species of each genus for which the data were available.


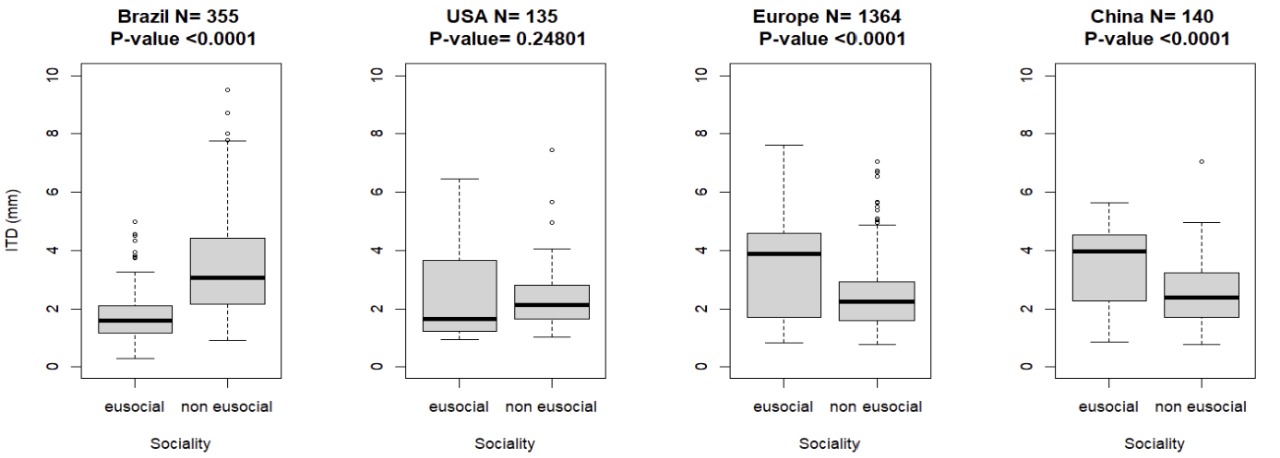


**Figure S2.** Comparisons of ITD across regions for each sociality class solely with species level information. P-values presented were obtained with a log likelihood ratio test.
